# Effects of Glutamine, Curcumin and Fish Bioactive Peptides Alone or in Combination on Intestinal Permeability in a Chronic-Restraint Stress Model

**DOI:** 10.3390/ijms24087220

**Published:** 2023-04-13

**Authors:** Ludovic D. Langlois, Sarah Oddoux, Kanhia Aublé, Paul Violette, Pierre Déchelotte, Antoine Noël, Moïse Coëffier

**Affiliations:** 1 Univ Rouen Normandie, Inserm, ADEN UMR1073 “Nutrition, Inflammation and Microbiota-Gut-Brain Axis”, F-76000 Rouen, France; 2 Laboratoire DIELEN, F-50110 Tourlaville, France; 3 Nutrition Department, CHU Rouen, F-76000 Rouen, France

**Keywords:** intestinal permeability, stress, glutamine, curcumin, fish bioactive peptides

## Abstract

Irritable bowel syndrome (IBS), a multifactorial intestinal disorder, is often associated with a disruption in intestinal permeability as well as an increased expression of pro-inflammatory markers. The aim of this study was to first test the impact of treatment with glutamine (Gln), a food supplement containing natural curcumin extracts and polyunsaturated n-3 fatty acids (Cur); bioactive peptides from a fish protein hydrolysate (Ga); and a probiotic mixture containing *Bacillus coagulans*, *Lactobacillus acidophilus*, *Lactobacillus gasseri* and *Lactobacillus helveticus*. These compounds were tested alone on a stress-based IBS model, the chronic-restraint stress model (CRS). The combination of Gln, Cur and Ga (GCG) was also tested. Eight-week-old C57Bl/6 male mice were exposed to restraint stress for two hours every day for four days and received different compounds every day one week before and during the CRS procedure. Plasma corticosterone levels were measured as a marker of stress, and colonic permeability was evaluated ex vivo in Ussing chambers. Changes in the gene expression of tight junction proteins (occludin, claudin-1 and ZO 1) and inflammatory cytokines (IL1β, TNFα, CXCL1 and IL10) were assessed using RT-qPCR. The CRS model led to an increase in plasma corticosterone and an increase in colonic permeability compared with unstressed animals. No change in plasma corticosterone concentrations was observed in response to CRS with the different treatments (Gln, Cur, Ga or GCG). Stressed animals treated with Gln, Cur and Ga alone and in combination showed a decrease in colonic permeability when compared to the CRS group, while the probiotic mixture resulted in an opposite response. The Ga treatment induced an increase in the expression of the anti-inflammatory cytokine IL-10, and the GCG treatment was able to decrease the expression of CXCL1, suggesting the synergistic effect of the combined mixture. In conclusion, this study demonstrated that a combined administration of glutamine, a food supplement containing curcumin and polyunsaturated n-3 fatty acids, and bioactive peptides from a fish hydrolysate was able to reduce colonic hyperpermeability and reduce the inflammatory marker CXCL1 in a stress-based model of IBS and could be of interest to patients suffering from IBS.

## 1. Introduction

Irritable bowel syndrome (IBS) is a functional gastro-intestinal disorder, now called a disorder of the gut–brain axis (DGBA), with a high prevalence of around 11% of the population worldwide [1] or 5% in France [2]. IBS is characterized by gastrointestinal symptoms, including abdominal pain, bloating, diarrhea and/or constipation, that have negative impacts on quality of life and contribute to a high prevalence of depression [3]. IBS also has considerable socio-economic consequences and represents a major proportion of gastrointestinal workloads in both primary and secondary care [4,5].

IBS is a multifactorial disorder that is likely caused by environmental factors (food and gastrointestinal infections), psychosocial factors (stress and anxiety) and gut physiology (low-grade inflammatory responses, abnormal gut–brain responses and gut microbiota alteration). Patients with IBS frequently show visceral hypersensitivity as well as epithelial barrier dysfunction, which contributes to pain and defecation symptoms. Low-grade chronic inflammation has been proposed in patients with IBS [6] with alterations in circulating or mucosal cytokines [7]. The role of gut barrier disruption has also been discussed. Interestingly, Zhou et al. reported that patients exhibiting visceral hypersensitivity had increased intestinal permeability [8]. In particular, intestinal hyperpermeability mainly occurs in patients with diarrhea-predominant IBS (IBS-D). Tight-junction protein expression or localization in epithelial cells was altered both in the small intestinal and colonic mucosae [9] of patients with IBS. Again, occludin expression was negatively associated with the visceral hypersensitivity score [10]. It is well established that inflammatory factors may contribute to the alteration of gut barrier functions [7]. In addition, patients with IBS exhibit gut microbiota dysbiosis. Fecal microbiota transplantation from patients with IBS into mice is associated with altered motility, increased intestinal permeability and anxiety-like disorders [11]. Targeting gut barrier functions and gut microbiota might be an interesting strategy to limit abdominal pain in patients with IBS.

Stress appears to play a key role in IBS development or maintenance. IBS patients are more sensitive to experimental stress, resulting in higher cortisol blood levels [12], and they display hypothalamic–pituitary–adrenal axis dysregulation. In humans, stress induced by public speech was associated with increased intestinal permeability [13]. Thus, to mimic IBS, animal models of stress are often used (neonatal stress, restraint stress, water avoidance stress or chronic unpredictable mild stress) and show increased intestinal permeability [14,15].

To improve IBS symptoms, we selected four compounds based on their capacity to target gut microbiota, gut barrier function and inflammatory and stress responses: glutamine, curcumin, bioactive peptides from fish protein hydrolysate and probiotics. Several experimental data underline the ability of glutamine to limit intestinal hyperpermeability [14,16,17]. Interestingly, patients with IBS exhibit a decrease in the colonic expression of glutamine synthetase [18]. In a randomized controlled trial, glutamine supplementation was found to be associated with improvements in IBS symptoms and a limitation of intestinal permeability in patients with post-infectious IBS was found [19], suggesting that glutamine may be of interest in patients with non-infectious IBS. Curcumin, extracted from Curcuma longa, exhibits anti-inflammatory and anti-oxidant properties. Curcumin showed beneficial effects in an experimental model of colitis [20]. However, in a recent meta-analysis, Ng et al. concluded that current findings are limited, and clinical studies should be encouraged for the use of curcumin in patients with IBS, which appears to be safe and well-tolerated [21]. In recent decades, the use of protein lysates from various food sources has seen increasing interest because of their biological activities. Bioactive peptides from a fish protein hydrolysate, named Gabolysat^®^, exhibited anxiolytic effects in a conditional burying test [22] and are able to modulate stress responses by acting on the pituitary–adrenal axis, on sympathoadrenal activity and on gamma-aminobutyric acid content [23]. In addition, the combination of Gabolysat^®^ with magnesium and vitamin B6 has also been proven to be efficient with respect to adjusting anxiety disorders [24]. Finally, probiotics show interesting effects, but due to the variety of the available species, strains, and doses, and duration and other conditions, making recommendations for probiotics remains difficult. Recently, Colomier et al., nicely reviewed different probiotics that have been tested in randomized controlled trials according to targeted gastro-intestinal symptoms [25].

In the present study, we thus aimed to evaluate the effectiveness of selected compounds (glutamine, curcumin, bioactive peptides from fish protein hydrolysate Gabolysat^®^ and probiotics) to limit low-grade colonic inflammatory responses and colonic hyperpermeability in mice using a model of chronic-restraint stress (CRS) by administering them alone and in combination.

## 2. Results

### 2.1. Response to Stress

#### 2.1.1. Acute-Restraint Stress Model

**Figure 1:**
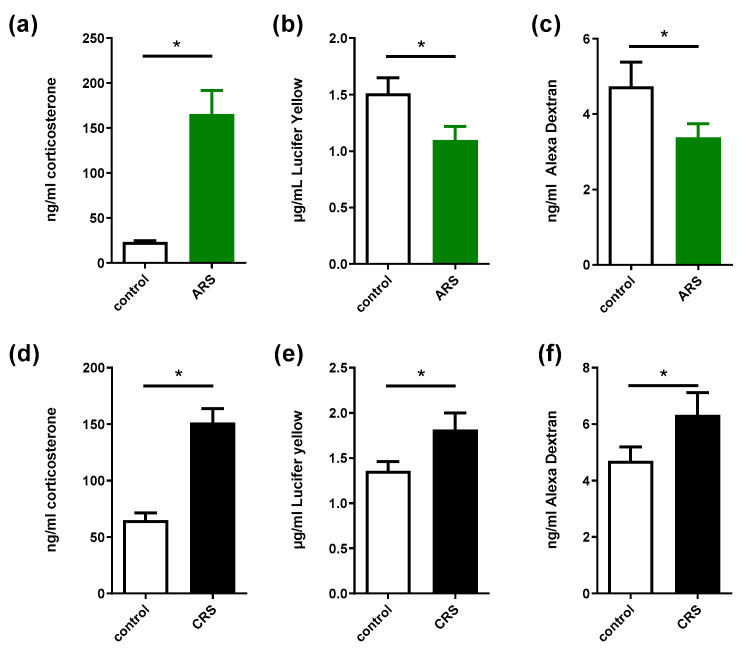
Plasma corticosterone levels and colonic permeability in response to acute (ARS) or chronic (CRS) stress models. (**a**,**d**): plasma corticosterone levels; (**b**,**e**) Lucifer yellow levels; (**c**,**f**): Alexa Dextran concentrations measured in Ussing chambers in control vs. ARS or CRS groups, respectively. Data are presented as means ± SEM. *n* = 8–20 per group. *, *p* < 0.05, Student’s *t*-test.

The level of corticosterone in the control group was 23.01 ng.mL^−1^. ARS resulted in a significant increase in plasma corticosterone levels compared to the control group (7.3-fold change, Figure 1a). In addition, colonic permeability decreased, as demonstrated by the decrease in both Alexa Dextran and Lucifer yellow concentrations in the serosal compartment of the Ussing chambers (Figure 1b,c).

#### 2.1.2. Chronic-Restraint Stress Model

The control group’s level of corticosterone was 64.84 ng.mL^−1^. An increase in plasma corticosterone was observed in the CRS group compared to the control group (2.2-fold change, Figure 1d). Contrary to ARS, CRS induced an increase in both Lucifer yellow and Alexa Dextran levels in the Ussing chamber (Figure 1e,f), showing that this model induces an increase in colonic permeability. However, we did not observe significant changes in inflammation-related gene expressions, as well as tight-junction protein gene expression (Supplementary Figure S1).

### 2.2. Evaluation of Plasma Corticosterone and Intestinal Permeability in Treated and Stressed Mice

**Figure 2:**
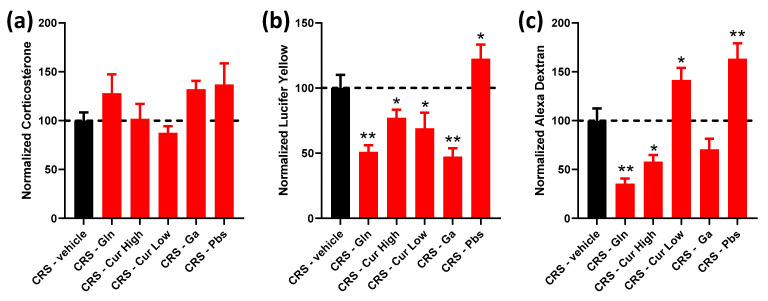
Effect of each selected compound administered individually on plasma corticosterone levels and colonic permeability. Normalized corticosterone levels (**a**) measured in plasma and Lucifer yellow (**b**) and Alexa Dextran (**c**) concentrations measured in Ussing chambers in CRS-vehicle (black bars) vs. CRS-Glutamine (Gln), CRS-Curcuméga^®^ (Cur) at high and low doses, CRS-Gabolysat^®^ (Ga) and CRS-Probiotics (Pbs) groups (red bars). *n* = 8–20 per group. Data are presented as means ± SEM. Data were compared by one-way ANOVA with Dunnett’s post hoc tests; *, *p* < 0.05 and **, *p* < 0.01 vs. CRS-vehicle. Dashed line represents the level observed in untreated CRS mice.

**Figure 3:**
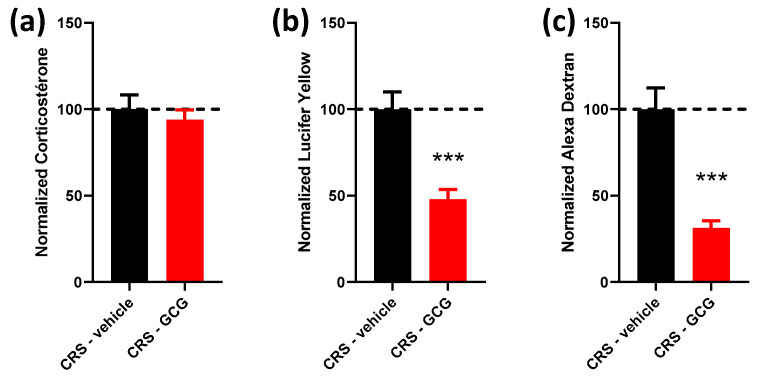
Effect of combined selected compounds, glutamine, Curcuméga^®^ and Gabolysat^®^ (GCG), on plasma corticosterone levels and colonic permeability. Corticosterone levels (**a**) measured in plasma and Lucifer yellow (**b**) and Alexa Dextran (**c**) concentrations measured in Ussing chambers in the CRS-vehicle (black bars) vs. CRS-GCG group (red bars). *n* = 10–19 per group. Data are presented as means ± SEM. ***, *p* < 0.001, Student’s *t*-test. Dashed line represents the level observed in untreated CRS mice.

**Figure 4:**
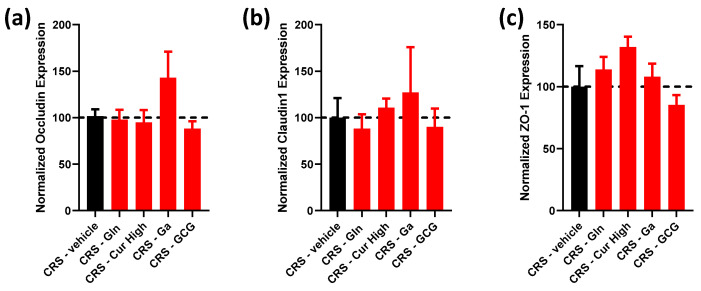
Effect of each selected compound administered individually and the GCG mix on colonic tight-junction protein gene expression. (**a**) Normalized occludin, (**b**) claudin-1 and (**c**) ZO-1 gene expression in CRS (black bars) vs. CRS-Glutamine (Gln), CRS-Curcuméga^®^ (Cur) at high and low doses, CRS-Gabolysat^®^ (Ga) and CRS-GCG groups (red bars). *n* = 7–16 per group. Data are presented as means ± SEM. Data were compared by one-way ANOVA with Dunnett’s post hoc tests. Dashed line represents the level observed in untreated CRS mice.

**Figure 5:**
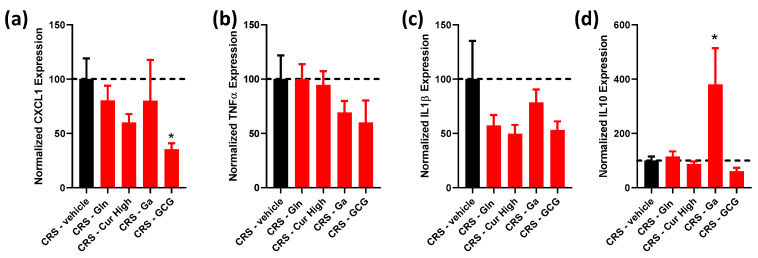
Effect of each selected compound administered individually and the GCG mix on colonic inflammation-related gene expression. (**a**) Normalized CXCL1, (**b**) TNFα, (**c**) IL1β and (**d**) IL10 gene expression in CRS (black bars) vs. CRS-Glutamine (Gln), CRS-Curcuméga^®^ (Cur) at high and low doses, CRS-Gabolysat^®^ (Ga) and CRS-GCG groups (red bars). *n* = 4–18 per group. Data are presented as means ± SEM. Data were compared by one-way ANOVA with Dunnett’s post hoc tests (**a**–**c**) or Kruskal–Wallis with Dunn’s post hoc tests (**d**). *, *p* < 0.05 vs. CRS-vehicle. Dashed line represents the level observed in untreated CRS mice.

In all following figures (Figure 2, Figure 3, Figure 4 and Figure 5), data from treatment groups are normalized to the stress group, which was set to 100%. Regarding plasma corticosterone levels, no significant change was observed between all groups (glutamine, Curcuméga^®^ (Laboratoire DIELEN, Tourlaville, France) at low and high doses, Gabolysat^®^ (Laboratoire DIELEN) and probiotics) (Figure 2a). Overall, each treatment induced a decrease in LY (457 Da) colonic permeability compared to the stressed controls (Figure 2b), except for the probiotics that were associated with an increase in LY colonic permeability (1.96-, 1.29-, 1.44- and 2.10-fold change for Gln, Curcuméga^®^ at high and low doses and Ga, respectively). The AD (3 kDa) colonic permeability was only significantly reduced after Gln and Curcuméga^®^ at high doses (2.81- and 1.72-fold change, respectively), while Curcuméga^®^ at low doses and probiotics enhanced AD colonic permeability compared to the CRS-vehicle group (Figure 2c). For the Gabolysat^®^ treatment, a trend of decrease was observed, but the difference did not reach significance (*p* = 0.087, Figure 2c).

Taking previous results into account, we then evaluated the effects of combined glutamine, Curcuméga^®^ and Gabolysat^®^ (GCG). Again, the plasma corticosterone level remained unchanged between the CRS-vehicle and CRS-GCG groups (Figure 3a). However, both LY and AD colonic permeabilities were significantly reduced compared to stressed untreated mice (2.08- and 3.19-fold change, respectively, Figure 3b,c), and this was the most effective reduction in AD colonic permeability that was observed in our experiments.

We then evaluated tight-junction-related gene expression in these different groups. As shown in Figure 4, we did not observe significant modifications in occludin, claudin-1 and ZO-1 mRNA levels in the colonic mucosa.

### 2.3. Evaluation of Inflammation-Related Gene Expression in the Colonic Mucosa of Treated Stressed Mice

To evaluate the colonic inflammatory response, we evaluated the gene expression of pro-inflammatory (CXCL1, TNFα and IL1β) and anti-inflammatory (IL10) cytokines (Figure 5a–c). For pro-inflammatory cytokines, we only observed a significant effect for CXCL1 (p (Kruskal–Wallis) = 0.0302). CXCL1 is a chemokine that is considered one of the functional homologs of IL-8 in humans, since the gene encoding for IL-8, *cxcl8*, is lacking in rodents [26,27]. Changes observed for TNFα and IL1β remained insignificant. Interestingly, post-tests revealed that the CXCL1 mRNA level was significantly reduced only in the CRS-GCG group compared to that in the CRS-vehicle group (Figure 5a), suggesting the synergistic effect of Gln, Curcuméga^®^ and Gabolysat^®^ bioactive peptides on this parameter.

Concerning the anti-inflammatory cytokine IL10, CRS-GA exhibited an increase in IL10 mRNA levels (Figure 5d) that was not observed in other treated groups, including the CRS-GCG group.

## 3. Discussion

In this study, we (i) validated our CRS model as a model of IBS by showing its ability to increase colonic permeability; (ii) tested the effects of four different compounds on plasma corticosterone levels, colonic permeability and the modulation of tight-junction-related and inflammation-related gene expression; and (iii) tested the combination of the effective treatments.

First, we showed that our protocol of ARS was an effective stressor, as demonstrated by the large increase in plasma corticosterone levels. Interestingly, ARS induced a decrease in permeability that was reversed when stress was repeated in the CRS model. Stress in rats can induce transient analgesia [28] in response to acute water avoidance stress. This effect is mediated by CRF receptor 2 activation. In our model, it is possible that acute stress-induced decreased permeability could be linked to this occurrence of analgesia and share similar molecular pathways. The repetition of ARS for four days was able to induce an increase in colonic permeability measured with two different fluorophores, which returned values that were differ from one another in terms of molecular weight. The 3 kDa Alexa Dextran and the smaller 457 Da Lucifer yellow used in this study showed similar changes in response to stress and the different administered treatments, suggesting an alteration in paracellular flux in the colonic region. The CRS protocol used in this study had moderate effects on mRNA-level coding for tight-junction proteins and inflammatory cytokines in mice colons compared to control animals, which could be related to the relatively short time period (4 days) tested in this model. Restraint stress, although mostly used as wrap-restraint stress, is an established IBS model [29]. Interestingly, in the present study, some treatments modified the response to the CRS model.

Second, glutamine treatment combined with CRS induced a decrease in colonic permeability, as demonstrated by the reduction in the concentrations of both Alexa Dextran and Lucifer yellow in the Ussing chambers when compared to the CRS-vehicle group. Curcuméga^®^ treatments had similar effects, except in the low-dose group in which the Alexa Dextran levels increased. The effect of glutamine and curcumin has already been supported by other studies in the context of IBS [14,17,30]. In addition, glutamine has been shown to enhance the beneficial effect of a low-FODMAP diet in IBS patients [31] However, Curcuméga^®^ does not only contain curcumin but also polyunsaturated n-3 fatty acids and vitamins E/D3, which may contribute to the beneficial effects of Curcuméga^®^ on gut barrier functions. Indeed, in a chronic unpredictable stress model, EPA and DHA supplementation was able to prevent the alteration of intestinal integrity [32]. Similarly, the limitation of intestinal barrier disruption has also been reported after vitamin E or D3 supplementation in experimental models [33]. We can thus speculate that Curcuméga^®^ effects were related to the mixture of these compounds. The CRS group treated with Gabolysat^®^ bioactive peptides also showed a decrease in intestinal permeability that was significant only with the Lucifer yellow probe. Thanks to its lower size, the Lucifer yellow probe appears to be more sensitive with respect to measuring paracellular flux in Ussing chambers [34]. The Gabolysat^®^ treatment was used for the first time in a stress-based IBS model and showed some promising results. Fish bioactive peptides have been shown to potentially attract interest in preventing visceral damage in gastric injury models in rats [35] and have anxiolytic effects [22]. In the present study, we reported that Gabolysat^®^ could also help by maintaining colonic mucosal barrier integrity.

In contrast, the probiotic mixture showed opposite effects compared to the other treatments by further increasing permeability in response to stress. The specific strains used in this study—*Bacillus coagulans*, *Lactobacillus acidophilus*, *Lactobacillus gasseri* and *Lactobacillus helveticus*—could have played a role in the controversial effect shown in this CRS model. Although the selected bacteria all showed beneficial effects in IBS patients [36,37,38,39], we used, in the present study, generic strains. Reports of the unfavorable effects of probiotics are rare, but these were made in some clinical trials. For instance, another specific strain, *Lactiplantibacillus plantarum* MF1298, has also been shown to have deleterious effects on IBS by increasing the severity of IBS symptoms [40], but the mechanisms have not been deciphered. It would thus be of interest to study the mechanisms underlying the increase in intestinal permeability observed in the present work with this specific combination of probiotics in future experiments. Based on these results, the probiotic mixture was not included in the combined treatments in the last set of experiments in order to avoid suppressing the beneficial effects of the three other treatments administered to the CRS animals.

Finally, the mixed treatment including glutamine, Curcuméga^®^ and Gabolysat^®^ (GCG) showed the most pronounced decrease in intestinal permeability and decreased the mRNA level of the inflammatory marker CXCL1, which was not observed with each compound administered alone. CXCL1 and its receptor CXCR2 play a key role in the recruitment of neutrophils and thus regulate colonic mucosal inflammation [41], and CXCL1 is considered a homolog of IL-8, which is not present in rodents [26,27]. These results suggest that a possible synergistic effect occurs with the combination of treatments. Interestingly, a recent study by Faucher et al., evaluated the potential beneficial effect of glutamine, curcumin and the probiotic *Lacticaseibacillus rhamnosus* GG alone and combined in a chronic unpredictable mild-stress mice model [42]. The synergistic beneficial effect of the three different treatments on anxiety and depression-like behaviors was demonstrated, as assessed by elevated plus maze and tail suspension tests. The combined treatment used in the latter study [42] had two compounds in common with the GCG treatment, reinforcing the idea of the synergistic action of the different treatments in our model and suggesting a potential anxiolytic and anti-depressive effect. As the CRS model is known to cause anxiety-like behavior [43], it would be interesting to measure the effect of GCG treatments in a behavioral test such as the elevated plus maze test. Further experiments would be required to dissect the molecular mechanisms engaged by each treatment in order to test whether or not the different compounds act via converging pathways and whether their effects are additive or if different molecular pathways are activated and work together in decreasing intestinal permeability.

The present study has some limitations. One of the limitations of this study is the fact that Curcuméga^®^, Gabolysat^®^ bioactive peptides and probiotic treatments are composed of several different compounds, peptides or bacterial strains. Thus, the specific effect of each component of these treatments could not be determined in our study. Further examinations would be required to deepen our understanding of the molecular pathways involved in these effects. In addition, the treatments were administered before and during chronic-restraint stress. We were not able to determine whether the beneficial effects were preventive or curative. Finally, we evaluated intestinal permeability in Ussing chambers by focusing on occludin, claudin-1 and ZO-1 proteins. It could be of interest to assess other tight-junction proteins and perform immunohistochemistry in further investigations.

In conclusion, we demonstrate in the present study that the combined administration of glutamine, Curcuméga^®^ and Gabolysat^®^ bioactive peptides is more effective for reducing gut barrier disruption and inflammatory responses than each compound alone is. Further investigations should evaluate the effects of this approach to reduce clinical gastro-intestinal symptoms in patients with intestinal disorders.

## 4. Materials and Methods

### 4.1. Animals

Animal care and experimentation complied with both French and European Community regulations and followed ARRIVE guidelines [44], and the experiments were approved by the Regional Ethical Committee (CENOMEXA, the Ethics Committee of Normandy for Animal Experimentation, acceptance number #6686–2016071512471751 v8). For this study, 7-week-old C57BL/6JRJ mice were purchased from Janvier Labs (Genest-St.-Isle, France) and acclimated to the animal facility (with a controlled temperature (20 ± 2 °C) and a 12 h light–dark cycle) for 1 week before experimentation. Animals were given ad libitum access to food and water and were placed in standard home cages (5 mice/cage).

### 4.2. Chronic-Restraint Stress (CRS)

The mice were briefly anesthetized with isoflurane and placed in restraint cages (Bioseb^®^, Vitrolles, France) for two hours before returning to their home cage. For acute-restraint stress (ARS), restraint stress was induced one time. For chronic-restraint stress (CRS), the restraint sessions were repeated for four consecutive days (at the same hour) before sacrificing the animals. Control mice were kept in their home cage during the procedure.

### 4.3. Treatments

All treatments were provided daily for seven days before starting the stress procedure and were maintained during the stress experiments.

Glutamine (Gln) was diluted in drinking water to provide 2 g·kg^−1^ of body weight per day. The Gln solution was prepared and replaced every day.

Curcuméga^®^ was administered by oral gavage so that the dose of curcumin received was 500 or 100 mg.kg^−1^.day^−1^ depending on the group. Curcuméga^®^ also contains polyunsaturated n-3 fatty acids, vitamin E and vitamin D3. For 500 mg.kg^−1^ of curcumin, the mice also received 80 mg.kg^−1^ of EPA, 50 mg.kg^−1^ of DHA, 12 mg.kg^−1^ of Vitamin E and 5 µg.kg^−1^ of Vitamin D3 daily. The Curcuméga^®^ solution was diluted in corn oil to obtain the desired concentration. Corn oil was thus used as the vehicle treatment in the control groups.

The Gabolysat^®^ bioactive peptides from the fish protein hydrolysate (Ga) were administered daily at a dose of 30 mg.kg^−1^ by oral gavage, while control mice received water as the vehicle.

Based on the literature, we selected four bacteria species provided by THT^®^ (Isnes, Belgium): *Bacillus coagulans* (ATCC7050/LMG6326), *Lactobacillus acidophilus* (LMG8151), *Lactobacillus gasseri* (LMG26661) and *Lactobacillus helveticus* (LMG26307). The probiotic mixture (Pbs) containing 25% of each strain was administered at 1 × 108 CFU by oral gavage once a day, and control animals received maltodextrins as the vehicle.

Dose selection was based on previous animal studies showing the beneficial effects of each treatment [17,29,35,45]. The equivalent mouse doses were calculated from human recommendations or rat doses when relevant [46].

### 4.4. Euthanasia and Sampling

The mice were briefly anesthetized with isoflurane and decapitated. Blood samples were collected right after the animals’ decapitation in blood collection tubes and centrifuged (4 °C, 1650 G, 15 min), and plasma was frozen at −80 °C. Samples of a fresh colon were collected to evaluate paracellular permeability in the Ussing chambers. The remaining colonic samples were collected, washed with ice-cold PBS, immediately frozen in liquid nitrogen and stored at −80 °C for further analyses.

### 4.5. Colonic Permeability in Ussing Chambers

Colonic permeability was assessed by measuring both the Lucifer yellow (LY, 457 Da) and Alexa 680-dextran (AD, 3000 Da) fluxes in the Ussing chambers which had an exchange surface of 0.07 cm^2^ (Harvard Apparatus, Holliston, MA, USA). The samples were maintained at a temperature of 37 °C. Both fluorophores were placed on the mucosal side, and the medium from the serosal side was collected after 3 h and stored at −80 °C. The fluorescence level of Lucifer yellow (excitation 428 nm, emission 540 nm) and Alexa Dextran (excitation 665 nm, emission 710 nm) in a serosal medium was measured in a 96-well black plate with a Spark^®^ multimode microplate reader (Tecan, Männedorf, Switzerland). The values were converted to concentrations using a standard curve.

### 4.6. RT-qPCR

Mucosal total RNA was extracted from samples as previously described [47]. After reverse transcription of 1 μg of total RNA into cDNA by using 200 units of SuperScript™ II Reverse Transcriptase (Life Technologies, Cergy-Pontoise, France), qPCR was performed by SYBR™ Green technology on a BioRad CFX96 real-time PCR system (BioRad Laboratories, Marnes la Coquette, France). GAPDH was used as the endogenous reference gene. The specific primers are displayed in Table 1. We focused on gene-encoding tight-junction proteins (ZO-1, occludin and claudin-1) and pro-inflammatory (CXCL-1, TNFα and IL-1β) and anti-inflammatory (IL-10) markers.

### 4.7. Corticosterone Assay

Plasma corticosterone levels were measured using a commercially available ELISA kit (Abnova^®^ KA0468, Abnova, VWR international SAS, Fontenay-sous-Bois, France) by following the instructions provided by the manufacturer.

### 4.8. Statistical Analysis

Data were analyzed using the GraphPad Prism 8.3 software (GraphPad Software Inc., San Diego, CA, USA) and expressed as mean ± standard error. Values were compared by ANOVA with Dunnett’s post hoc tests or Kruskal–Wallis with Dunn’s post hoc tests if data distribution departed from normality. Results were considered significant when the *p*-value was lower than 0.05.

## Figures and Tables

**Table 1:** Sequences of primers used for qPCR.

Gene	Forward or Reverse	Primer Sequence	Primer Length	Amplicon Length	GenBank Accession Nb
Claudin-1	F	CTGGGTTTCATCCTGGCTTC	20	443	NM_016674
Claudin-1	R	TTGATGGGGGTCAAGGGGTC	20		
Occludin	F	AGACTACACGACAGGTGGGG	20	124	NM_008756
Occludin	R	CTGCAGACCTGCATCAAAAT	20		
ZO-1	F	GCAGACTTCTGGAGGTTTCG	20	194	NM_009386
ZO-1	R	CTTGCCAACTTTTCTCTGGC	20		
TNFα	F	TGTCTACTCCTCAGAGCCCC	20	166	NM_013693
TNFα	R	TGAGTCCTTGATGGTGGTGC	20		
IL1β	F	CCCAAAAGATGAAGGGCTGC	20	169	NM_008361
IL1β	R	AAGGTCCACGGGAAAGACAC	20		
CXCL1	F	ACTCAAGAATGGTCGCGAGG	20	84	NM_008176
CXCL1	R	GGGACACCTTTTAGCATCTTTTGG	24		
IL10	F	ACCTGGTAGAAGTGATGCCC	20	153	NM_010548
IL10	R	GCTCCACTGCCTTGCTCTTAT	21		
GAPDH	F	CATCACTGCCACCCAGAAGA	20	318	NM_001289726
GAPDH	R	AAGTCGCAGGAGACAACCT	19		

## Data Availability

Not applicable.

## Supplementary Materials

The following supporting information can be downloaded at: https://www.mdpi.com/article/10.3390/ijms24087220/s1.
