# Supplementary material for: Effects of Glutamine, Curcumin and Fish Bioactive Peptides Alone or in Combination on Intestinal Permeability in a Chronic-Restraint Stress Model
Source: Int J Mol Sci. 2023 Apr 13;24(8):7220. doi: 10.3390/ijms24087220 (PMC10139227; doi:10.3390/ijms24087220)
Supplement: Supplementary file 1 [file ijms-24-07220-s001.zip › ijms-2306006-supplementary.pdf]

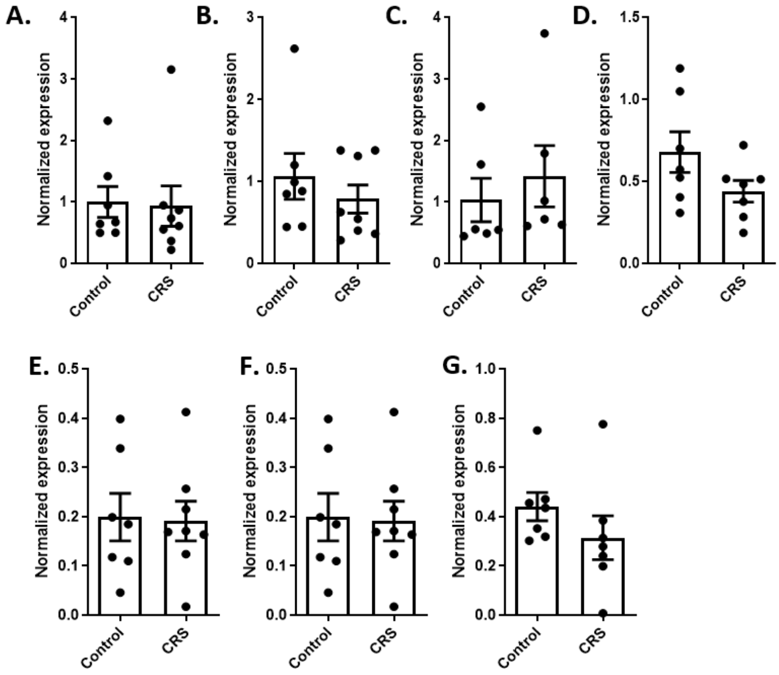

Figure S1. Effect of CRS on colonic inflammation related proteins and tight junction proteins gene expression (A) CXCL1, (B) TNFα, (C) Il1β and (D) IL10, (E) Occludin, (F) Claudin 1 and (G) ZO-1 gene expression in control vs. CRS groups. n = 7-8 per group.
